# Prediction of One‐Dimensional Metallicity and π‐Band Superconductivity in Rhodizonate Radical Pancakes

**DOI:** 10.1002/anie.202507041

**Published:** 2025-09-25

**Authors:** Alvaro Lobato, Fernando Izquierdo‐Ruiz, Martin Rahm

**Affiliations:** ^1^ Department of Chemistry and Chemical Engineering Chalmers University of Technology Gothenburg SE‐412 96 Sweden; ^2^ Present address: Faculty of Chemistry Department of Physical Chemistry Complutense University of Madrid Madrid Spain 28040

**Keywords:** Chemical bonding, Organic conductors, Organic superconductors, Oxidation states, Oxocarbon anions, Structure prediction

## Abstract

Computational exploration of condensed phases made of potassium and carbon monoxide leads to predictions of stable salts composed of cyclic six‐membered oxocarbon anions and K^+^ cations, K*_n_* (C_6_O_6_)*_m_*. The states of reduction in these systems are wide‐ranging, with C_6_O_6_ molecules formally reduced by −2, −3, −3.5, and −6 in semiconducting and metallic phases. Special attention is paid to K_3_C_6_O_6_, in which triply charged radical anions stack closely and equidistantly in one dimension. Equidistant interactions of radicals are exceedingly rare and typically unstable due to spontaneous symmetry breaking, Peierls or Jahn–Teller distortion. The predicted exception of K_3_C_6_O_6_ is explained by inter‐ring multicenter bonding, also known as pancake bonding, in combination with large ionic repulsion. This fascinating interplay of interactions facilitates an exceptionally high density of states at the Fermi level and leads us to predictions of metallicity, a negative temperature coefficient of resistivity, and rare π‐band superconductivity. These predictions reinvigorate the search for new organic conductors and superconductors using molecular design of metallic salts.

## Graphical abstract

Equidistant stacking of radicals is typically unstable due to symmetry‐lowering distortions. We predict that such arrangements can be stabilized by multicentered covalent (“pancake”) bonding and Coulombic repulsion between negatively charged radicals. This leads to design principles for a new class of organic conductors—potentially superconductors—based on abundant, environmentally benign elements.

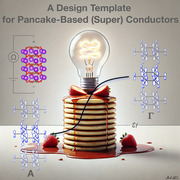

## Introduction

In this computational study, we address materials composed formally of K and CO and focus especially on K_3_C_6_O_6_, a material we predict to feature oxocarbon radical anions, metallicity, and the potential for rare π‐band superconductivity. Our study is in part motivated by the possibility of producing metallic organic salts, i.e., compounds where ionic and covalent bonding situations are mixed in a metallic solid‐state structure.^[^ ^1^, ^2^ ^]^ Such compounds can exhibit a range of properties needed in, for example, spintronics, superconductors, optoelectronic devices, and electrochemical sensors.^[^ ^3^, ^4^, ^5^ ^]^

CO is a common ligand useful for probing electronic structure in part due to its ability to engage in π‐backdonation. However, CO can also react with itself. The thermodynamic ground state of CO in the condensed phase is experimentally uncertain. Predictions (at *T* → 0 K) indicate that the ground state is not molecular but akin to a lactone‐like polymer.^[^ ^6^, ^7^ ^]^ A variety of CO‐based polymeric structures, both metallic and narrow band gap semiconductors, have been observed at elevated pressures.^[^ ^8^, ^9^ ^]^

The reactivity of CO is increased by reduction, which allows it to undergo homologation reactions,^[^ ^10^ ^]^ forming oxocarbon anions.^[^ ^11^, ^12^, ^13^, ^14^, ^15^ ^]^ Such processes constitute potent C─C coupling routes and synthesis of oxycarbon‐based materials have been pursued for over a century. The first attempts were probably made by Berzelius, Wohler, and Kindt^[^ ^16^ ^]^ in 1823, who reacted potassium hydroxide with carbon to form dipotassium croconate (K_2_C_5_O_5_). Around the same time, Liebig^[^ ^17^ ^]^ circulated CO through molten potassium and made what was much later identified as a mixture of potassium ethynediolate and potassium benzenehexolate. Since then, a variety of s‐, p‐, d‐, and f‐block elements have been used to reduce CO, yielding different oxocarbon compounds (see, e.g., Ref. [10] and references therein).

Some oxocarbon compounds are stabilized by extensive π‐conjugation,^[^ ^18^, ^19^, ^20^ ^]^ and anions tend to adopt cyclic structures with all carbon atoms bonded to a carbonyl or an enolic oxygen atom. Interest in such materials has various motivations: Materials such as oxocarbon squarate, Li_2_C_4_O_4_, are promising anode materials.^[^ ^21^ ^]^ Others, such as M_2_C_6_O_6_ (M = Li, Na, and K), are ultra‐high capacitors^[^ ^22^, ^23^ ^]^ and cathode materials.^[^ ^24^ ^]^ Pb_3_C_6_O_6_ is a potential photovoltaic material,^[^ ^25^, ^26^ ^]^ while M(C_6_O_6_) (M = Fe, Co, Mn),^[^ ^27^ ^]^ Mn_5_(C_6_O_6_)_2_,^[^ ^28^ ^]^ and Cu_3_(C_6_O_6_)_2_^[^ ^29^, ^30^ ^]^ are examples of narrow‐band‐gap metal–organic frameworks.^[^ ^27^, ^29^, ^31^ ^]^ Some oxocarbon anions facilitate the production of hydrocarbons though Fischer–Tropsch‐type processes.^[^ ^11^, ^12^, ^13^, ^14^, ^15^ ^]^

Several studies have focused on the properties of these molecular anions, including, for example, how the aromaticity (or antiaromaticity) of anions may vary with charge.^[^ ^32^, ^33^, ^34^ ^]^ In contrast, relatively little attention has been paid to the bonding situation in the solid state, where ionic, covalent, and van der Waals interactions can combine to create unique extended chemical structures (see, e.g., Refs. [35, 36, 37]).

In what follows, we outline predictions of the solid‐state phase diagram of K*_n_* (CO)*_m_* at ambient conditions of pressure. We then examine the unusual *P*6/*mmm* ground state of K_3_C_6_O_6_, which we predict to be highly ionic, metallic, and potentially superconducting. Finally, we propose K_3_C_6_O_6_ as a design template for a family of conductors and superconductors and outline rationales for engineering their electronic structure.

## Results and Discussion

**Figure 1:**
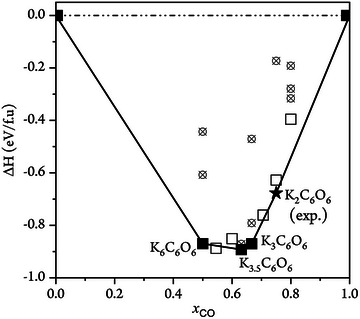
Convex hull of the K:CO system showing formation enthalpies at *T* → 0 K and ambient conditions of pressure. Structures predicted to be of lowest enthalpy at a given stoichiometry are indicated by filled squares for stable phases and open squares for thermodynamically or dynamically unstable or metastable phases. Crossed circles indicate higher enthalpy structures. The experimentally known *Fddd* phase^[^ ^38^ ^]^ of K_2_C_6_O_6_ is indicated by a black star.

We rely on structure prediction algorithms coupled to large‐scale density functional theory (DFT) calculations to explore the possible chemistry between K and CO. The procedures underlying these efforts are detailed in the computational methods section of the Supporting Information. Our predictions of the thermodynamic stability of various K:CO stoichiometries are summarized by a convex hull diagram in Figure 1. In this representation, stable compounds lie on the solid line, whereas those phases that are unstable or metastable with respect to decomposition into neighboring stoichiometries lie above the line. Our reference of pure CO, i.e., where *x*_CO_ = 1 in Figure 1, is a chain‐like *Pna*2_1_ phase identical to the predictions by Xia et al.^[^ ^7^ ^]^ Structural details and phonon band structures of all ground‐state phases used to construct the convex hull are provided in Sections S1–S4 of the Supporting Information.

Our calculations predict a clear preference for homologation of CO into a variety of molecules and extended phases that are stable with respect to elemental K and pure CO. These structures range from one‐ and three‐dimensional polymers to three‐, four‐, and six‐membered oxocarbon anion rings (see Figure S2 for some selected examples of these structures). Compounds in which molecular CO remains distinct as a ligand coordinated to K are here exclusively calculated to be unstable or metastable with respect to decomposition into other phases. We note, however, that Wu et al. have detected eight‐coordinate carbonyl complexes with Ca, Ba, and Sr in low‐temperature matrix isolation experiments.^[^ ^39^, ^40^, ^41^ ^]^

Six‐membered oxocarbon anions are predicted to be the most thermodynamically favored, as was also calculated by Yamashita et al. with Na as counterions,^[^ ^36^, ^37^ ^]^ and Liam et al.^[^ ^42^ ^]^ We refer to these six‐membered cyclic C_6_O_6_ *^n^* ^−^ anions as rhodizonates but note that they could alternatively be called cyclohexanehexoneates, hexaketocyclohexaneates, triquinoylates, or tetrahydroxybenzoquinones (THBQs). Several rhodizonate‐based salts have been previously synthesized (see, e.g., Refs. [21, 22, 23, 24, 25, 26, 27, 28, 29, 30, 31]), and we will return to discuss some of them in what follows.

**Figure 2:**
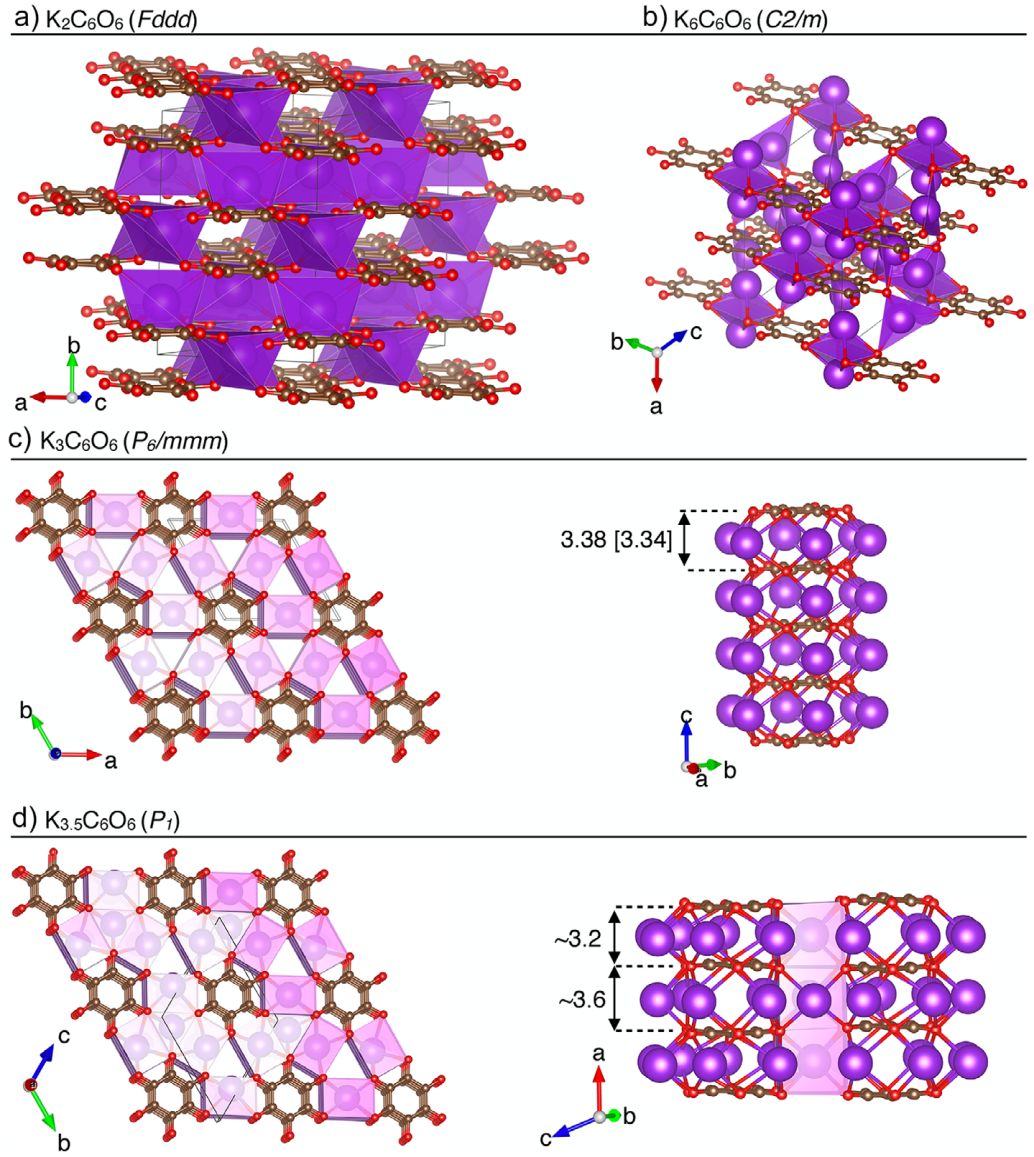
Unit cells of the a) experimental *Fddd* phase of K_2_C_6_O_6_ (from Ref. [38]), b) predicted *C*2/*m* phase of K_6_C_6_O_6_, c) predicted equidistantly stacked *P*6/*mmm* phase of K_3_C_6_O_6_, d) predicted nonequidistantly stacked *P*1 phase of K_3.5_C_6_O_6_. K, C, and O atoms are represented as purple, brown, and red spheres, respectively. Indicated distances in Å are calculated at the PBE‐D3(BJ) [HSE06‐D3(BJ)] levels of theory.

All ground states featured on the K–CO convex hull shown in Figure 1 are based on differently charged rhodizonate anions (Figure 2a–d). Rhodizonates have a remarkable ability to hold different charges, and while a formal charge of −2 is most common in experimental structures, −4 and −3 have also been inferred.^[^ ^28^, ^30^, ^31^ ^]^ One of these, the *Fddd* phase of K_2_C_6_O_6_, is the only pure K‐oxocarbon material in the Cambridge structural database^[^ ^38^ ^]^ (Figure 2a). Because of its size (28 atoms in the primitive cell), the *Fddd* phase could not be identified by our structure search. However, and encouragingly, our predicted lowest energy phase for the same stoichiometry calculates as only 48 meV f.u.^−1^ (1.1 kcal mol^−1^) above the experimental structure (Figure 1). We note that whereas our structure search has been extensive, it cannot be exhaustive, in part due to practical limitations in the sizes of unit cells described. Nonetheless, any missing *ground state* phase is likely to also feature rhodizonate ions.

In the *Fddd* phase of K_2_C_6_O_6_, formally doubly charged (C_6_O_6_)^2−^ rhodizonates orient in layers (Figure 2a). The anions in this experimental structure are slightly distorted from planarity with a C─C─C─C dihedral angle of 10.94°, an angle in good agreement with our DFT predictions of 12.27°. The K^+^ counterions are situated in‐between layers of anions in such a way as to closely coordinate six oxygens. The identification of what is formally (C_6_O_6_)^2−^ and K^+^ ions is confirmed by a topological^[^ ^43^ ^]^ analysis of the electron density (Table 1), which indicates a highly ionic bonding contribution in this material. Our calculations reveal that this phase is a semiconductor with a band gap of 1.2 eV (Figures 2 and S6), a possible underestimation due to the level of theory.

**Table 1:** Partial charges of atoms and molecular fragments in identified ground state structures.^a)^

	K_2_C_6_O_6_ (*Fddd*)	K_3_C_6_O_6_ (*P*6/*mmm*)	K_3.5_C_6_O_6_ (*P*1)	K_6_C_6_O_6_ (*C*2/*m*)
K	+0.87	+0.86	+0.84	+0.81
C	+0.83	+0.74	+0.70	+0.42
O	−1.12	−1.16	−1.19	−1.23
(C_6_O_6_)	−1.74	−2.58	−2.94	−4.86

^a)^ Bader charges predicted from the topology of the electron density using the quantum theory of atoms in molecules (QTAIM).

On the other side of the hull in Figure 1 sits the predicted *C*2/*m* phase of K_6_C_6_O_6_ (Figure 2b). This material is remarkable in terms of the large charges it permits on the relatively small rhodizonate ions. Our estimate of −4.9 (Table 1) is close to the formal reduction by six electrons provided by a stochiometric amount of K. We emphasize that such highly charged organic anions are exceedingly rare, even exceeding the alkali metal −4 reduction of corannulene.^[^ ^44^, ^45^ ^]^ The (C_6_O_6_)^6−^ rhodizonates in this phase are subtly distorted from planarity: we predict them to be D_2_ symmetric and feature two different C─C distances (1.443 and 1.442 Å) and a C─C─C─C dihedral angle of 2.32°. Rhodizonates in the *C*2/*m* phase arrange in rows with each highly charged anion bridged by two K^+^ cations (Figure 2b). The different rows of anions are offset relative to each other such that the bridging cations of one row also coordinate exactly in the middle of a neighboring anion. The band gap of this semiconducting phase exceeds 1.5 eV (Figure S3).

The remaining two identified ground states, a *P*6/*mmm* phase of K_3_C_6_O_6_ (Figure 2c) and a *P*1 phase of K_3.5_C_6_O_6_ (Figure 2d), are both predicted to be metallic and feature perfectly planar rhodizonates that are stacked without any cations in‐between them. The *P*6/*mmm* phase of K_3_C_6_O_6_ is the focus of the remainder of this work and we will return to discuss its close structural relationship to the *P*1 phase of K_3.5_C_6_O_6_.

### The Unusual Structure of K_3_C_6_O_6_

The *P*6/*mmm* phase of K_3_C_6_O_6_ (hereafter only K_3_C_6_O_6_) contrasts sharply with all other compounds identified on the K–CO phase diagram, and it even conflicts with chemical expectations. The rhodizonates are in this material formally radicals carrying three negative charges (−2.6 by our analysis, cf., Table 1). The anions are predicted to be perfectly planar, *D*_6_ *_h_* symmetric, with equidistant C─C (1.46 Å) and C─O (1.27 Å) bonds. Instead of arranging themselves in an alternating fashion with respect to their K^+^ counterions, these rhodizonates prefer to be close, stacking face‐to‐face directly and *equidistantly* on top of each other (Figure 2c). One‐dimensional chains of radicals are expected to be unstable with respect to a spontaneous symmetry breaking.^[^ ^46^ ^]^ Such deformations, which occur to avoid degeneracy of electronic levels, are known as Peierls and Jahn–Teller distortions in physics and chemistry, respectively.

The equidistant structure we predict in K_3_C_6_O_6_ does not appear to be an artifact of the level of theory. The inter‐ring distances in K_3_C_6_O_6_ are calculated as 3.38 Å at the PBE‐D3(BJ) level and closer still, 3.34 Å, in a HSE06‐D3(BJ) calculation. This distance is slightly below 2 *r*_vdw_(C) (Figure S10), which is noteworthy considering it is between triply charged (hence electrostatically repelling) anions. The effect of the van der Waals correction in our calculations is relatively small: it lowers the formation enthalpy of K_3_C_6_O_6_ by 40 meV (ca 5%) relative to K(s) and CO(s), and it is responsible for an inter‐ring shortening of 0.09 Å. The K^+^ counterions are in this structure cubically coordinated to oxygen and are positioned in concentric rings around each stack of anions (Figure 2c).

Phonon calculations at hybrid‐DFT level are computationally prohibitive for us due to the dense *k*‐point sampling required. Nevertheless, HSE06‐D3(BJ) relaxation starting from symmetry‐broken starting point configurations returns to the equidistant stacking. 1D constrained scans of the inter‐anionic separation in a 1 × 1 × 2 supercell at both r^2^SCAN + rVV10 and HSE06‐D3(BJ) levels also locate a minimum at the equidistant geometry (Figure S11). That our level of theory is capable of converging to nonequidistant stackings of rhodizonates is evidenced by the *P*1 phase of K_3.5_C_6_O_6_ (Figure 2d). In the latter phase, symmetry is broken into alternating pairs of rhodizonates, still organized in 1D stacks (Figure 2d).

We note that while K_3_C_6_O_6_ has been previously predicted by Lian et al.,^[^ ^42^ ^]^ the phase has only been discussed in terms of its potential performance as an anode (K^+^ storage) material, while its metallic nature and unusual structure have been overlooked. Lian et al. have also predicted a different phase of K_3.5_C_6_O_6_ with *Fmmm* symmetry, which features dimeric stacking of rhodizonates. We have identified the *Fmmm* phase in our structure search but predict it to lie 0.1 eV f.u.^−1^ above the *P*1 phase.

To the best of our knowledge, there are only a few reported structures of rhodizonate salts in which the anion *might* carry a charge of −3. The crystal structures of MnRbC_6_O_6_ and MnCsC_6_O_6_ both feature face‐to‐face stacking of distorted (nonplanar) anions,^[^ ^28^ ^]^ wherein in the latter structure the closest carbon atoms of adjacent anions are only 2.69 Å apart. The reported MOFs FeC_6_O_6_, and Cu_3_(C_6_O_6_)_2_ similarly contain dimers of distorted anions only 2.85 and 2.75 Å apart, respectively.^[^ ^30^, ^31^ ^]^ An important distinction from our predictions of K_3_C_6_O_6_ is that these experimental structures do not feature equidistant stacking of anions. These experimental structures, furthermore, all contain some amount of water, and they are all based on transition metal cations that appear to induce magnetic interactions (we will discuss magnetism in a later section).

Why do some rhodizonates neighbor one another despite such glaringly unfavorable electrostatics? And why do these anions, which are radicals, resist both Peierls and Jahn–Teller in K_3_C_6_O_6_ to align equidistantly instead of pairing up? Conventional face‐to‐face π‐stacking interactions are not strong enough to overcome the electrostatic repulsion of neighboring highly charged anions.^[^ ^47^, ^48^ ^]^ In fact, π‐interactions (between closed‐shell ring systems) are only weakly attractive or repulsive and typically result in T‐shaped or parallel displaced configurations.^[^ ^49^, ^50^ ^]^

### Electronic Structure of K_3_C_6_O_6_

**Figure 3:**
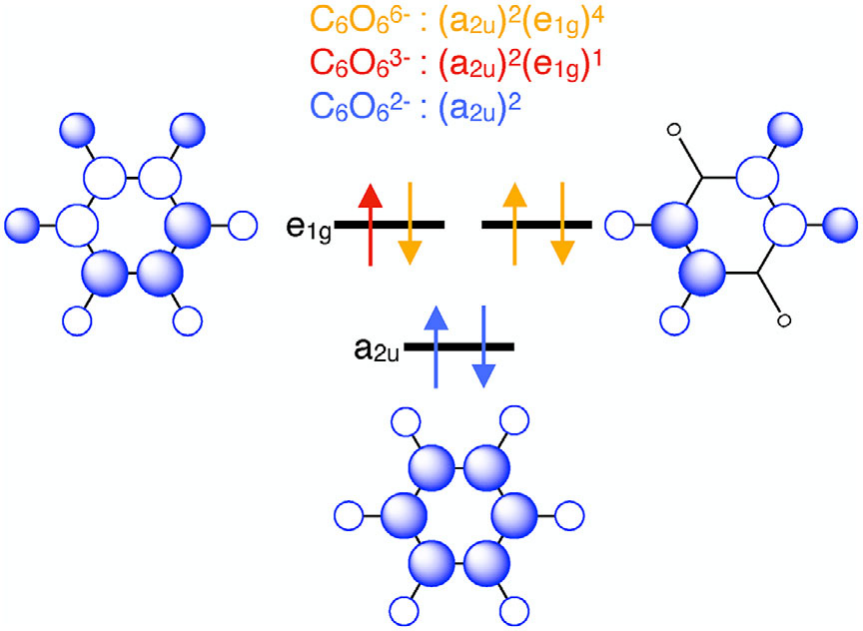
Top view of frontier (π)–MOs of isolated rhodizonates, (C_6_O_6_)*^n^* ^−^. Electronic configurations are shown for (C_6_O_6_)^2−^ in blue, (C_6_O_6_)^3−^ in red and (C_6_O_6_)^6−^ in gold. The singly occupied e_1g_ orbital of the (C_6_O_6_)^3−^ anion is key to understanding the structural and electronic properties of K_3_C_6_O_6_.

To understand the preference for face‐to‐face stacking of rhodizonates carrying a charge near −3, we first look at the highest occupied molecular orbital (MO) levels of the isolated anion. The π–MO ordering in the neutral (*D*_6_ *_h_*) C_6_O_6_ molecule has been extensively studied theoretically (see, e.g., Refs., [32, 51, 52, 53]). Figure 3 shows a schematic subset, the highest occupied MOs, which suffices to highlight the major electronic differences between the rhodizonates we study. Whereas (C_6_O_6_)^6−^ and (C_6_O_6_)^2−^ are both closed‐shell anions, the singly occupied e_1g_ molecular orbital (SOMO) of the (C_6_O_6_)^3−^ radical anion permits us to rationalize some of the unusual electronic—and through them structural—properties of K_3_C_6_O_6_.

**Figure 4:**
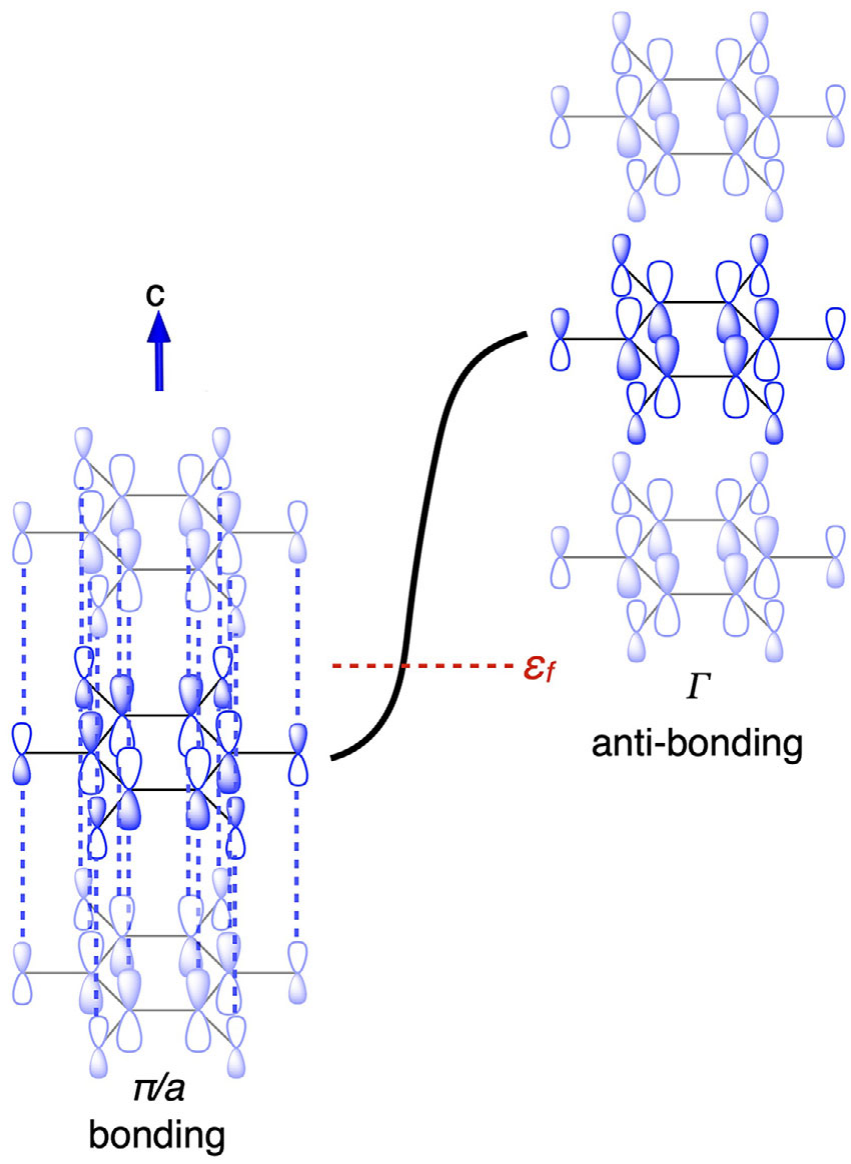
Intermolecular multicenter bonding explains the face‐to‐face stacking of triply charged rhodizonates. Figure depicts one of two degenerate frontier π‐bands formed from the e_1g_ orbitals shown in Figure 3 running in one dimension. The (e_1g_)^1^ origin of these bands ensures a ¼‐band occupation and predominantly  inter‐anion bonding levels below the Fermi level, *ε*_F_. Dashed blue lines highlight bonding interactions between C and O on neighboring anions. K^+^ ions are omitted for clarity. This sketch is illustrative of all paths through the 1st Brillouin zone of the *P*6/*mmm* space group that corresponds to the real space lattice vector c, i.e., the anion stacking direction. Examples of such directions in K_3_C_6_O_6_ are A → Γ, H → K, and L → M in later figures. However, in the real material the frontier π‐bands are only ¼‐occupied on average. Figure S9 provides an example of how weaker bonding interactions orthogonal to the stacking direction can give rise to flatter band regions.

**Figure 5:**
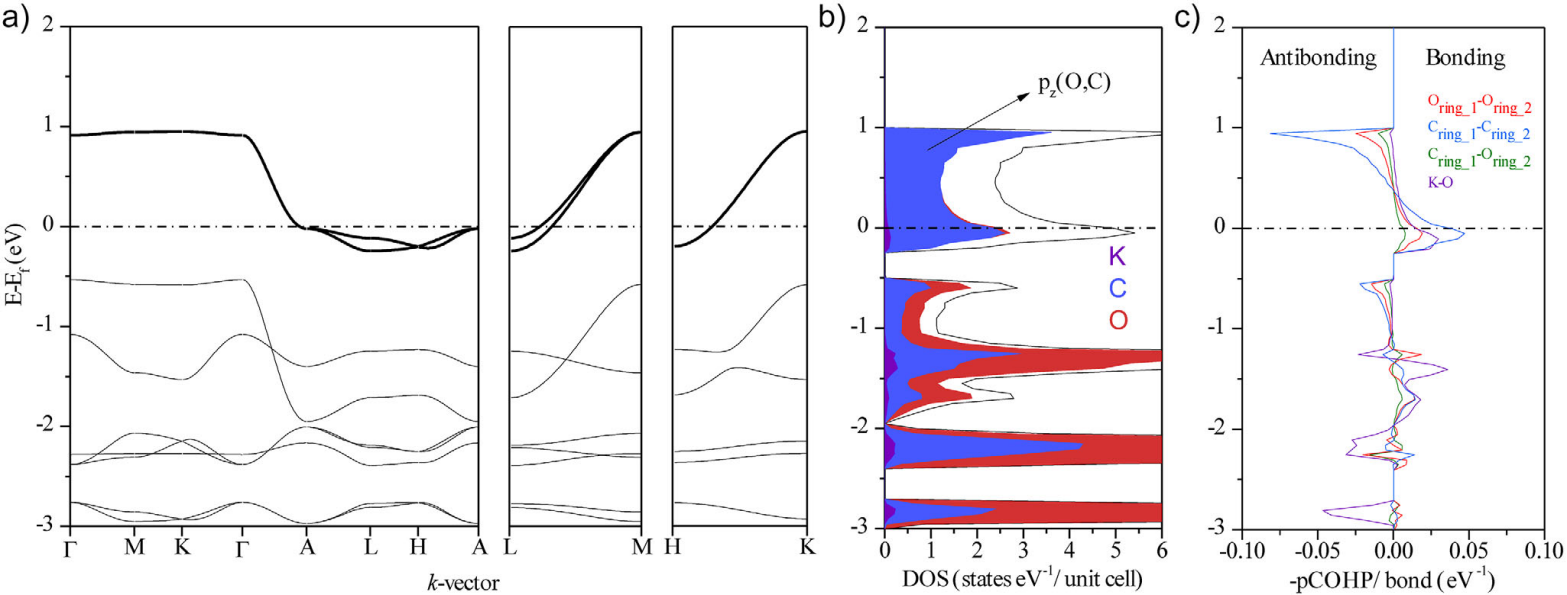
Electronic structure analysis of K_3_C_6_O_6_. a) band structure b) DOS near the Fermi level (*ε*_F_). Orbital projected DOS are shown for K (purple), C (blue), and O (red). c) COHP bonding analysis with anion‐anion interactions denoted as C─C (blue), C─O (green), and O─O (red). A bonding K─O interaction (purple) is also present near the Fermi level.

Figure 4 explains how the e_1g_ SOMO of (C_6_O_6_)^3−^ shown in Figure 3 can spread into highly dispersed π‐bands along the stacking direction of the rhodizonates, and how their partial occupation yields predominantly bonding character below the Fermi level, *ε*_F_. Our band‐structure calculation (Figure 5a) shows that these two frontier π bands are degenerate along Γ─A, Γ─K, M─K, and K─H but split elsewhere in the Brillouin zone and are on average quarter‐filled, consistent with the (e_1g_)^1^ configuration of the (C_6_O_6_)^3−^ anion. Although a single component of the e_1g_ SOMO (Figure 3) appears symmetry‐lowered, the degenerate pair and, in the solid, the occupied bands together yield a charge density that respects the full *P*6/*mmm* symmetry. The *D*_6_ *_h_* geometry of the ring follows from the rhodizonate being centered on a 6/*mmm* (*D*_6_ *_h_*) site.

Density of state (DOS) calculations predict K_3_C_6_O_6_ to be distinctly metallic (Figure 5b). And as expected, the DOS near the Fermi level share a distinct similarity to that we expect from a 1D system.^[^ ^54^, ^55^ ^]^ This electronic structure gives rise to clear peaks at the band edges at Γ, A, L, M, H, and K, which explain the sizable DOS just below the Fermi level. In line with common condensed‐matter usage, we refer to these as van Hove singularities, i.e., DOS features arising from band extrema or saddle points, regardless of whether they represent strict mathematical divergences in this quasi‐1D system.

The type of bonding we describe in Figures 4 and 5 is a kind of intermolecular multicenter bonding but infinitely extended along the face‐to‐face stacking direction of rhodizonate anions. The closest molecular analogy to this kind of extended interaction is the sharing of electrons between face‐to‐face stacked planar radicals, often referred to as pancake bonds.^[^ ^56^, ^57^, ^58^, ^59^ ^]^

Pancake bonds are distinct from van der Waals interactions and conventional π‐stacking both in terms of structure and bond strength.^[^ ^60^ ^]^ These kinds of spin‐pairing interactions are covalent in nature and are formed between planar radicals with a ring‐to‐ring distance smaller than the sum of van der Waals radii of neighboring atoms. Our crystal orbital Hamilton population (COHP) analysis shown in Figure 5c indicates substantial inter‐rhodizonate bonding, with an integrated COHP of ∼0.44 eV (∼10 kcal mol^−1^) for nearest‐neighbor contacts. This value is, possibly fortuitously, of the same order as reported stabilization energies for strong pancake‐bonded dimers,^[^ ^56^ ^]^ which range from 0.52 to 0.78 eV (12–18 kcal mol^−1^). We note, however, that ICOHP magnitudes are not bond‐dissociation energies but track bonding strength trends (see methods section). The rhodizonates we study are oriented further apart (∼3.3 Å) compared to the shortest molecular pancake bonds, ca. 2.93 Å.^[^ ^61^ ^]^ We ascribe the larger spacing to the high −3 charge of neighboring radicals, which both increases electrostatic repulsion and the effective van der Waals contact distance, and to the need to accommodate K⁺ coordination in the extended lattice.

Pancake bonding has almost exclusively been discussed in terms of dimers of π‐delocalized systems, such as tetracyanoquinodimethane or phenalenyls.^[^ ^56^, ^57^, ^58^, ^59^ ^]^ In the solid state, the structural diversity is richer and pancake‐bonded systems can be structurally characterized as either i) dimers in which the radicals exhibit slight distortions from planarity or ii) equidistant face‐to‐face stacking of planar radicals, i.e., similar to the case we predict in K_3_C_6_O_6_. However, equidistant stacking is rare^[^ ^59^, ^62^, ^63^, ^64^, ^65^ ^]^ and such examples are in general composed of neutral radicals (or diradicals, e.g., in compounds containing CN_2_E_2_ [E = S, Se] groups)^[^ ^66^ ^]^ that are semiconducting. Aside from the rhodizonate‐based materials already mentioned (which are distorted and dimeric),^[^ ^21^, ^22^, ^23^, ^24^, ^25^, ^26^, ^27^, ^28^, ^29^, ^30^, ^31^, ^38^, ^42^ ^]^ the closest experimentally known analog to our predicted *P*6/*mmm* phase of K_3_C_6_O_6_ is probably potassium tetracyanoquinodimethane (K^+^TCNQ^•−^).^[^ ^67^ ^]^ The radicals in K^+^TCNQ^•−^are equidistantly stacked above 396 K, but undergo a Peierls distortion (dimerization) below this temperature. Salts of TCNQ are important historically, as they helped trigger a substantial interest in organic conduction in the 1970s and onwards.^[^ ^68^ ^]^

### Is K_3_C_6_O_6_ Metallic?

Two features make K_3_C_6_O_6_ stand out among molecular conductors. First, equidistant stacking of (C_6_O_6_)^3−^ yields quarter‐filled, highly dispersive, predominantly bonding π‐bands along the stacking direction (Figures 4 and 5). Second, the Fermi‐level DOS is unusually large. *N*(ε_F_) calculates as 0.19–0.27 states per eV per atom, depending on the level of theory (Figure S3), approaching elemental Cu (0.29) and exceeding some phonon‐mediated superconductors (e.g., MgB_2_ = 0.24^[^ ^69^ ^]^). Such high values, are to the best of our knowledge, unprecedented for a molecular solid.

The high values of *N*(ε_F_) derive from the peaked shape of the DOS. Such DOS topologies are rare and reminiscent of what can be found in some high‐pressure materials such as the 150 GPa *I* *m*3*m* phase of SH_3_.^[^ ^70^ ^]^ Nature tends to abhor such electronic features at ambient conditions of pressure, where they are usually avoided through structural symmetry breaking that causes the formation of electronic gaps or pseudo gaps. For example, many TCNQ‐based salts develop stack distortions that open gaps and yield semiconducting behavior. Even in TCNQ systems with reduced distortion, reported conductivities are typically modest, up to 1·10^−2^ S cm^−1^ at high temperature.^[^ ^71^, ^72^ ^]^ Semiconducting rhodizonate‐based MOFs, such as FeC_6_O_6_, likewise show conductivities in the mS range or below near ambient conditions.^[^ ^31^ ^]^ In contrast, both K_3_C_6_O_6_ and the *P*1 phase of K_3.5_C_6_O_6_ are predicted to be metallic irrespective of whether they stack equidistantly or not.

While gapless conduction in quasi‐1D systems is uncommon, it is feasible: highly ordered metal–organic nanoribbons have demonstrated conductivities on the order of 10^4^ S m^−1^,^[^ ^73^ ^]^ and purple oxomolybdate bronze (Li_0.9_Mo_6_O_17_) is an inorganic example of a quasi‐1D conductor where metallicity persists despite strong correlations, exhibiting spin‐charge separation and Luttinger‐liquid behavior.^[^ ^74^ ^]^

Proceeding under the assumption that metallicity in K_3_C_6_O_6_ is robust–conferred by favorable frontier orbital topology (Figures 3, 4, 5)—we suggest that rhodizonates with a charge near −3 can be considered design templates for 1D conductors. Our prediction for *N*(ε_F_) is sensitive to levels of theory (Figure S8). Nevertheless, the distance between stacked anions offers a means for rationally tuning the electronic structure: increasing the separation reduces the π‐band dispersion, which sharpens and can shift the van Hove‐type features toward ε_F_, increasing *N*(ε_F_) (Figure 5b). Thermal expansion should therefore increase *N*(*ε*_F_), suggesting a negative temperature coefficient of resistivity (*dρ*/*dT* < 0). In other words, in transport measurements K_3_C_6_O_6_ may masquerade as a semiconductor. We note that this DOS‐driven trend competes with the usual increase of phonon scattering with temperature, so the net behavior is ultimately an experimental question.

Besides physical tuning, such as raising temperatures or subjecting the material to strain or pressure, electrical conductivity might, through the same argument, be modulated by replacing counter cations. For example, by substituting K^+^ for larger ions (e.g., Rb^+^, Cs^+^, or organic cations) higher values of *N*(ε_F_) are expected. We aim to explore this hypothesis in forthcoming work.

We acknowledge that predicting metallicity here is bold: strong electronic correlations or other effects beyond our treatment could, in principle, drive a Mott‐like instability. However, such transitions typically arise for narrow, weakly dispersive bands near half‐filling. In contrast, K_3_C_6_O_6_ features broad, inter‐anion bonding π‐bands at quarter filling. The highly ionic environment and the large polarizability of the triply charged rhodizonates should also enhance screening and disfavor localization. As with any quasi‐1D conductor, subtle long‐period or higher‐order lattice modulations at low temperature cannot be excluded. In such a situation, we expect a semimetal or small‐gap state emerging from the same frontier‐orbital manifold. Ultimately, the possibility of a minute gap is best assessed by low‐temperature transport and high‐resolution diffraction.

### A Note on Magnetism

Materials composed of equidistantly stacked planar radicals can exhibit long‐range magnetic order. Often, such order is antiferromagnetic and present in semiconducting phases, although ferromagnetic and diamagnetic states have been observed.^[^ ^59^, ^75^, ^76^, ^77^ ^]^ Our spin‐polarized calculations performed on a doubled unit cell of K_3_C_6_O_6_ predict the material to be nonmagnetic. However, accurately predicting subtle magnetic couplings is challenging. While we consider more detailed analysis of magnetism to lie outside the scope of this work, we emphasize the need for careful characterization of such properties following the anticipated synthesis of these materials.

### Electron–Phonon Coupling in K_3_C_6_O_6_

Electron–phonon coupling, the interaction between charge carriers and lattice vibrations, determines the temperature dependence of resistivity, modifies band dispersions, and can promote or compete with symmetry‐lowering distortions. It is also relevant for thermoelectric performance and superconductivity, which we will return to discuss.

**Figure 6:**
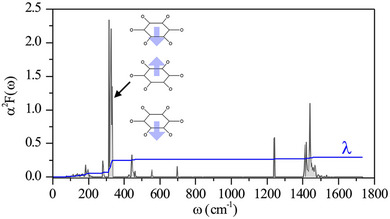
Frequency‐resolved Eliashberg spectral function α^2^F of the *P6/mmm* phase of K_3_C_6_O_6_ (gray) along with the cumulative contribution of the electron‐phonon coupling strength *λ* (blue line) calculated at the PBE‐D3(BJ) level. The majority of the electron–phonon coupling is associated with soft modes that alter distances between stacked rhodizonate anions.

The covalent nature and the sharpness of the DOS near the Fermi level in K_3_C_6_O_6_ suggest to us a potential for large electron–phonon coupling. To see why, we remind that the dispersion of the frontier π‐bands, and hence the number of states at the Fermi level are sensitive to the distance between rhodizonates. We can, therefore, anticipate electron–phonon coupling to be driven largely by low‐frequency motions of rhodizonates with respect to each other. Figure 6 shows the frequency‐resolved Eliashberg spectral function along with the cumulative electron–phonon coupling parameter, *λ*. As expected, a majority (two‐thirds) of the *λ* ≈ 0.3 value derives from inter‐ring displacement modes (at ∼330 cm^−1^) that couple to electrons involved in inter‐ring (“pancake”) bonding. That *λ* calculates as relatively small despite a large *N*(ε_F_) can be explained by the bonding character of the frontier π‐bands, which make them less sensitive to small inter‐ring displacements.

### Potential Superconductivity in K_3_C_6_O_6_

The unique structural and electronic properties of K_3_C_6_O_6_ lead us to consider it a potential superconductor. One reason^[^ ^78^ ^]^ behind this suspicion is the large number of states near the Fermi level, *N*(ε_F_). Electron–phonon coupling is the mechanism by which Cooper pairs are formed in conventional superconductors. Under this assumption, one can proceed to estimate a critical superconducting temperature, *T*_c_ of ∼40 mK (see the methods section). This low temperature corresponds to an estimated superconducting gap of ∼0.01 meV and a relatively low electron−phonon coupling constant *λ* of ∼0.3, which places this material in the weak coupling limit.

We note several caveats associated with our *T*_c_ estimate. First, the locations of the van Hove singularities responsible for the large DOS near the Fermi level depend on the exchange–correlation treatment (Figure S8). Second, numerical convergence is demanding. *T*_c_ depends on integrals over electronic crystal momenta (a *k*‐point grid) and phonon wavevectors (a *q*‐point grid), so practical grid sizes and thermal smearing introduce uncertainty (see Table S2 and Methods). Third, we evaluate *T*_c_ using the McMillan–Allen–Dynes approximation to isotropic Migdal–Eliashberg theory, with a fixed Coulomb pseudopotential and harmonic, adiabatic phonons; we have not included anharmonic renormalization or nonadiabatic/anisotropic effects. Organic superconductors are, furthermore, not always of the conventional (electron–phonon) variety,^[^ ^79^ ^]^ and there are reasons to suspect that other coupling mechanisms may be in play in a real material. Because rhodizonate anions can sustain multiple different charge states (Table 1), Cooper pair formation in K_3_C_6_O_6_ and related materials might, for example, be driven by redox lattice instabilities.^[^ ^79^ ^]^ Other possibilities include electronic coupling via polaronic or bipolaronic mechanisms^[^ ^80^, ^81^ ^]^ and polarization waves arising in a localized core–electron framework.^[^ ^82^ ^]^ The latter mechanisms, which we are not able to evaluate computationally, imply an importance of polarizability. Large organic anions of high charge are among the most polarizable material constituents possible. In other words, our stated *T*_c_ should be viewed as an order‐of‐magnitude baseline; more certain is the prediction of electron–phonon coupling dominated by inter‐ring modes near ∼330 cm^−1^ (Figure 6).

### Outlook: Toward Ionic π‐Based Superconductors

If K_3_C_6_O_6_ is metallic, it could serve as a template for discovering (and engineering) a new family of 1D π‐based superconductors. While our predicted *T*_c_ is low, the regime we consider—equidistant stacking of triply charged radical anions in an ionic solid—contrasts with typical organic molecular superconductors as well as intercalated graphite and 2D covalent π‐lattices.^[^ ^83^, ^84^ ^]^ Possible 2D superconductors such as LiC_6_,^[^ ^85^ ^]^ the experimentally established CaC_6_.^[^ ^86^ ^]^ and theoretically predicted phases including LiC_12_,^[^ ^83^ ^]^ AlC_8_,^[^ ^87^ ^]^ and HPC_3_^[^ ^88^ ^]^ indicate that intralayer π‐systems can couple to in‐plane vibrational phonon modes associated with covalent bonds, resulting in strong electron–phonon coupling and reported or predicted *T*_c_ values up to ∼30 K. K_3_C_6_O_6_ is different as superconductivity in it is predicted to be mediated through *intermolecular* (stacking), π–π modes rather than *intramolecular* covalent vibrations.

We attribute the low *T*_c_ calculated for K_3_C_6_O_6_ to two main factors: a low electron–phonon coupling strength *λ*, and a low average phonon frequency, ω. The mathematical relationships that connect *λ*, ω, and *N*(ε_F_) to *T*_c_ are provided elsewhere.^[^ ^78^, ^89^, ^90^ ^]^ What is essential for our discussion is that these parameters can be rationally altered (we want them to increase) through chemical and physical manipulation of K_3_C_6_O_6_. We wish to open the door to such manipulation by sharing a collection of rationales.

**Figure 7:**
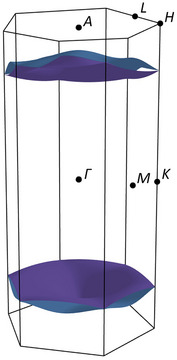
The Fermi surface of the *P*6/*mmm* phase of K_3_C_6_O_6_ is dominated by two frontier π‐bands that are degenerate in the direction of rhodizonate stacking (e.g., Γ–A, K–H). High‐symmetry points in the 1st Brillouin zone are indicated for reference. Modification of this surface by chemical or physical means may allow tailoring of electron–phonon coupling and superconductivity.

If superconductivity is present in K_3_C_6_O_6_, it will be intricately linked to a single pair of frontier π‐bands. This simplicity, which results from the quasi‐1D nature of the material, allows for relatively straightforward band engineering. The Fermi surface shown in Figure 7 is the subset of the frontier π‐bands that correspond to the highest occupied orbitals, resolved in reciprocal space. These levels are essential for superconductivity. The electron–phonon coupling strength, *λ*, reflects the average movement of these levels (or levels near them) upon phonon perturbation.^[^ ^78^, ^89^, ^90^ ^]^

Doping is one direct way of changing the electron–phonon coupling strength, *λ*, and we can qualitatively predict how without calculations. If K_3_C_6_O_6_ were to be *n*‐doped, its Fermi level would move up. The effects of this are clear from our COHP bonding analysis (Figure 5c): with *n*‐doping, the orbitals near the Fermi level become less bonding. At half‐filling, levels at the Fermi level would be effectively nonbonding, i.e., be equally bonding and antibonding. Such situations are characteristic of transition states and phase instabilities, and we therefore expect electron–phonon coupling to increase. If *n*‐doped K_3_C_6_O_6_ were to become unstable, it is likely to undergo a Peierls distortion that dimerizes the rhodizonates. Such dimerization would split the two frontier π‐bands into two pairs of bands and open a gap between them. The predicted *P*1 phase of K_3.5_C_6_O_6_ is one such example, in which (counted per dimerized unit cell) the lowest pair of π‐bands is ∼¾ filled, instead of one quarter as in K_3_C_6_O_6_. With such band filling, we expect the Fermi surface to be enriched by antibonding states (Figures 4 and 5a). Antibonding levels will naturally respond more strongly than bonding levels to changing orbital overlap and should therefore be more strongly coupled to low‐frequency phonon modes that compress and extend the rhodizonate stacks. Unfortunately, the ground state we predict for K_3.5_C_6_O_6_ is much too large and of too low symmetry to permit meaningful electron–phonon calculations by us. Experimentally, electrochemical gating, or controlled intercalation/deintercalation of alkali ions is plausible route to tune the filling without altering the stacking framework.

The rhodizonate stacking distance is a second parameter that can be directly controlled beside the band filling. As already mentioned, one way to do so is by ion substitution. Provided that the *P*6/*mmm* stacking motif is retained following such manipulation, the bonding character of the frontier bands will not change. However, increased rhodizonate distances should decrease their in‐stack bond strength, which should soften relevant inter‐ring phonon modes.

Increasing the average phonon frequency, ω, by chemical substitution is a third approach for engineering a higher *T*_c_ in K_3_C_6_O_6_‐derived materials. One way to effect such change is to select cations that contain strong covalent bonds to light elements, preferably hydrogen. Hydrogen‐containing cations could, in principle, couple high‐frequency motions to the frontier π‐bands of the rhodizonates, especially if hydrogen bonded.

Our suggestions for modulating conductivity and enhancing π‐based superconductivity are challenging to verify computationally, and we encourage experimental pursuit and characterization of these systems.

## Conclusion

The chemistry and history of oxocarbon materials are rich, both in terms of structure and their utility. Here, a thorough computational exploration of the K:CO phase diagram shows that six‐membered oxocarbon anions, rhodizonates, dominate the thermodynamic landscape. Rhodizonates possess a fascinating ability to hold different oxidation states, predicted to range from −2 in the semiconducting *Fddd* phase of K_2_C_6_O_6_ to −6 in the not‐yet‐made *C*2/*m* phase of K_6_C_6_O_6_, a likely record for small organic anions.

Interactions in the semiconducting phases appear dominated by electrostatics, with ions packed in an alternating fashion so as to maximize K–O interactions. In contrast, the predicted metallic *P*6/*mmm* phase of K_3_C_6_O_6_ and *P*1 phase of K_3.5_C_6_O_6_ exhibit face‐to‐face stacking of rhodizonates. The *P*6/*mmm* phase of K_3_C_6_O_6_ is especially unusual, even unique, for several reasons. Particularly noteworthy is the equidistant stacking of highly charged, i.e., electrostatically repelling, anions. Metallic organic salts are rare, and equidistant π‐stacking in them even more so.

We rationalize these structural and electronic features using molecular‐orbital and electrostatic arguments. The nodal structure of the e_1g_ SOMO of C_6_O_6_^3−^ is uniquely suited to extend into quarter‐filled, highly dispersive π‐bands with bonding character along the stacking direction. This multicenter interaction is reminiscent of “pancake” bonding, although equidistant stacking is commonly avoided by Peierls‐ or Jahn–Teller‐type distortions. In K_3_C_6_O_6_, however, the *T* → 0 K ground state appears to remain equidistant. We explain this structural feature by noting that any symmetry‐lowering modulation increases the repulsion between the −3 charged anions, providing a Coulombic penalty against Peierls‐like instabilities. Nevertheless, while we are confident in the predicted stability, we cannot exclude symmetry‐lowering instabilities that might emerge with methods beyond those employed here.

Density of states near the Fermi level of K_3_C_6_O_6_ is predicted to be exceptionally high, almost on par with elemental Cu and possibly exceeding that of superconductors like MgB_2_^[^ ^69^ ^]^ and high‐pressure SH_3_.^[^ ^91^ ^]^ This prediction is sensitive to the precise stacking distance, a parameter that will increase with temperature, leading us to expect a negative temperature coefficient of resistivity.

The quasi‐1D nature of K_3_C_6_O_6_ makes it especially suitable for band engineering, which otherwise can be challenging.^[^ ^92^ ^]^ We therefore suggest that K_3_C_6_O_6_ can act as a prototype for a new family of conductors and potentially superconductors. Such materials could be organic by suitable choice of counterion. While our prediction of a low critical superconducting temperature in the milli‐Kelvin range is uncertain, we outline a series of design rationales for increasing this temperature through physical and chemical modification of the number of states at the Fermi level, the average phonon frequency, and the electron–phonon coupling strength. We offer these predictions to encourage experimental synthesis, as well as further computational exploration.

## Supporting Information

Computational details, structural details, phonon spectra, atomic partial charges, DOS of selected low‐energy phases, and convergence with respect to thermal smearing. These materials are available in the Supporting Information of this article.

## Conflict of Interests

The authors declare no conflict of interest.

## Supporting information



Supporting Information

Supporting Information

## Data Availability

The data that support the findings of this study are available in the Supporting Information of this article.
