# Supplementary material for: Prediction of One‐Dimensional Metallicity and π‐Band Superconductivity in Rhodizonate Radical Pancakes
Source: Angew Chem Int Ed Engl. 2025 Sep 25;64(47):e202507041. doi: 10.1002/anie.202507041 (PMC12624317; doi:10.1002/anie.202507041)
Supplement: Supplementary file 1 — Supporting Information [file ANIE-64-e202507041-s001.pdf]

Supplementary Materials

Prediction of One-dimensional Metallicity and π-band Superconductivity in Rhodizonate Radical Pancakes

*Alvaro Lobato, Fernando Izquierdo-Ruiz, and Martin Rahm**

Department of Chemistry and Chemical Engineering, Chalmers University of Technology – SE-412 96 Gothenburg, Sweden.

*E-mail : martin.rahm@chalmers.se

S.1. Convex hull construction 3
S.2. Structural details of the reference structures 3
Pna2_1 phase of CO 3
Body centered cubic (I4/m-32/m) phase of elemental K 3
S.3.Structural details of relevant predicted structures for the convex hull construction 4
Fddd phase of $K_2C_6O_6$ optimized from the experimentally determined structure 4
P6/mmm phase of $K_3C_6O_6$ 4
P1 phase of $K_{3.5}C_6O_6$ 4
C2/m phase of $K_4C_6O_6$ 5
P_1 phase of $K_5C_6O_6$ 5
C2/m phase of $K_6C_6O_6$ 6
S.4. Selected examples of metastable structures 6
S.5. Density of States of Selected Low-Energy Phases 7
S.6. Phonon Spectra of Selected Low-Energy Phases 8
P6/mmm phase of $K_3C_6O_6$ 8
P1 phase of $K_{3.5}C_6O_6$. 8
C2/m phase of $K_4C_6O_6$ 8
P1 phase of $K_5C_6O_6$ 9
C2/m phase of $K_6C_6O_6$ 10
S.7. Partial charges of atoms and molecular fragments in identified structures 10
S.8. Density of States for the P6/mmm phase of $K_3C_6O_6$ 11
S.9 Inter-ring distances and van der Waals Radii Comparison in the P6/mmm phase of $K_3C_6O_6$ 12
S.10 1D Inter-ring distance potential Energy Surface 12
S.11. Convergence of T_c with respect to thermal smearing 12
S.12. Computational Details 13
S.11. References 14

S.1. Convex hull construction

Formation enthalpies of $K_xC_yO_y$ compounds are being calculated relative to ground state K and CO using the following formula:

$$\Delta H_f = \frac{H(K_xCO_y) - xH(K) - yH(CO)}{x+y}$$

For elemental potassium we use the body centered cube phase as a reference at zero pressure. In the case of CO, the experimental zero-pressure phase is predicted to be a disordered molecular structure called α-CO. However, recent calculations relying on structure prediction[1-2] predict polymeric structures as the ground states. We have independently predicted and evaluated a series of CO phases (Figure S1). Our analysis, yield the $Pna2_1$ chain-like structure predicted by Xia et al. as the most stable phase.

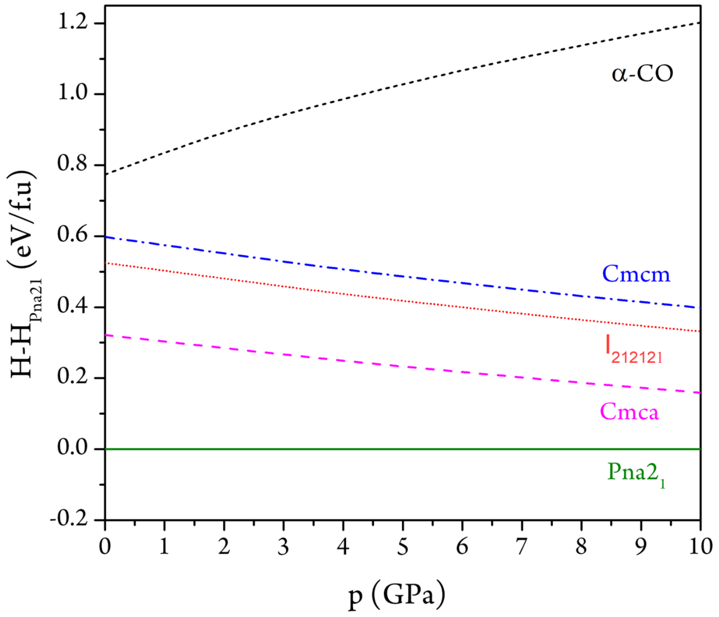

Figure S1. Enthalpy difference with respect to the $Pna2_1$ phase for different CO ground states between 0 and 10 GPa.

S.2. Structural details of the reference structures

$Pna2_1$ phase of CO

a = 10.36367, b = 4.191255, c = 8.164462. α = β = γ = 90.00

Atom	x	y	z	Wyckoff Position
C	0.22760	0.41233	0.33290	4a
C	0.12576	0.24416	0.25057	4a
C	0.12567	-0.09254	0.25072	4a
C	0.02468	0.41310	0.16718	4a
O	-0.06112	0.32023	0.08091	4a
O	0.31510	0.31875	0.41601	4a
O	0.22699	0.75561	0.30619	4a
O	0.02436	0.75531	0.19552	4a

Body centered cubic (I4/m-32/m) phase of elemental K

a = b = c = 5.15890, α = β = γ = 90.00

Atom	x	y	z	Wyckoff Position
K	0.00000	0.00000	0.00000	2a

S.3.Structural details of relevant predicted structures for the convex hull construction

All lattice parameters are expressed in Å

Fddd phase of $K_2C_6O_6$ optimized from the experimentally determined structure

a = 8.46650, b = 11.99918, c = 15.76531, α = β = γ = 90.00

Atom	x	y	z	Wyckoff Position
K	0.125	0.47432	0.125	16f
C	0.125	0.125	0.21907	16g
C	-0.02613	0.11927	0.17183	32h
O	0.125	0.125	0.29819	16g
O	0.846	0.10942	0.21065	32h

P6/mmm phase of $K_3C_6O_6$

PBE-D3: a = b = 8.22310, c = 3.37297. α = β = 90.00, γ = 120.00

Atom	x	y	z	Wyckoff Position
K	0.50000	0.00000	0.00000	3f
C	0.89727	0.79453	0.50000	6m
O	0.19227	0.38454	0.50000	6m

HSE-D3: a = b = 8.28267, c = 3.33950. α = β = 90.00, γ = 120.00

Atom	X	y	z	Wyckoff Position
K	0.50000	0.00000	0.00000	3f
C	0.89859	0.79717	0.50000	6m
O	0.18960	0.37921	0.50000	6m

P1 phase of $K_{3.5}C_6O_6$

a = 6.70860, b = 8.45778, c = 8.98826, α = 118.02, β = 111.79, γ = 90.03

Atom	x	y	z	Wyckoff Position
K	0.43017	0.91793	0.60678	1a
K	0.23471	0.45774	0.21493	1a
K	0.23476	0.98636	0.21486	1a
K	0.74752	0.93551	0.24038	1a
K	0.99265	0.98022	0.73122	1a
K	0.64122	0.1286	0.02817	1a
K	0.74728	0.53367	0.24018	1a
C	0.31841	0.57504	0.92093	1a
C	0.16803	0.5714	0.61585	1a
C	0.16815	0.27373	0.61599	1a
C	0.11084	0.37148	0.51376	1a
C	0.27426	0.37505	0.81901	1a
C	0.27417	0.67307	0.81888	1a
C	0.70227	0.27394	0.61633	1a
C	0.7022	0.57166	0.61627	1a
C	0.85758	0.57521	0.92128	1a
C	0.79955	0.67329	0.81932	1a
C	0.79964	0.37525	0.81938	1a
C	0.65722	0.37174	0.51411	1a
O	0.40831	0.66471	0.10028	1a
O	0.13094	0.65915	0.52698	1a

O	0.33332	0.28706	0.90758	1a
O	0.33323	0.84955	0.90731	1a
O	0.13116	0.09719	0.52724	1a
O	0.02119	0.28169	0.33417	1a
O	0.65035	0.09743	0.5276	1a
O	0.65024	0.65947	0.52749	1a
O	0.82927	0.28732	0.90803	1a
O	0.56724	0.28199	0.33452	1a
O	0.94721	0.66485	0.10064	1a
O	0.82915	0.84979	0.90793	1a

C2/m phase of $K_4C_6O_6$

Unstable with imaginary phonons: a = 5.71572, b = 14.62415, c = 5.62878, α = γ = 90.00, β = 101.71

Atom	x	y	z	Wyckoff Position
K	0.82465	0.34364	0.71700	8j
C	0.08203	0.08972	0.11107	8j
C	0.85269	0.00000	0.76709	4i
O	0.33058	0.33515	0.77401	8j
O	0.74188	0.00000	0.55088	4i

Modulated : a = 5.60626, b = 14.58588, c = 5.72940, α = γ = 90.00, β = 101.3256

Atom	x	y	z	Wyckoff Position
K	0.28441	0.65094	0.14340	4e
K	0.28170	0.16107	0.70611	4e
C	0.88936	-0.08765	0.89546	4e
C	0.88876	0.40878	0.44102	4e
C	0.23253	0.50269	0.64744	4e
O	0.22514	0.66802	0.63208	4e
O	0.22523	0.16120	0.20551	4e
O	0.44841	0.50593	0.75912	4e

P_1 phase of $K_5C_6O_6$

a = 5.39217, b = 7.21330, c =7.21821, α = 99.95, β = 82.48, γ = 97.52

Atom	x	y	z	Wyckoff Position
K	0.14736	0.93777	0.60358	1a
K	0.78177	0.08140	0.37645	1a
K	0.78191	0.62300	0.91779	1a
K	0.14723	0.39696	0.06304	1a
K	0.46553	0.50928	0.49072	1a
C	0.65659	0.11916	0.88096	1a
C	0.36772	0.09291	0.17792	1a
C	0.36788	0.82196	0.90707	1a
C	0.56112	0.19773	0.07325	1a
C	0.56126	0.92678	0.80238	1a
C	0.27231	0.90058	0.09930	1a
O	0.65193	0.37228	0.14960	1a
O	0.10536	0.79946	0.20025	1a
O	0.65209	0.85046	0.62791	1a
O	0.82342	0.22032	0.77997	1a
O	0.27711	0.64738	0.83078	1a
O	0.27692	0.16917	0.35242	1a

C2/m phase of $K_6C_6O_6$

a = 8.72682, b = 8.20270, c = 7.72401, α = γ = 90.00, β = 103.54

Atom	x	y	z	Wyckoff Position
K	0.15484	0.28363	0.64012	8j
K	0.17022	0.00000	-0.02687	4i
C	0.02966	0.34806	0.09538	8j
C	0.43767	0.00000	0.80889	4i
O	0.05819	0.20460	0.18531	8j
O	0.36575	0.00000	0.6322	4i

S.4. Selected examples of metastable structures

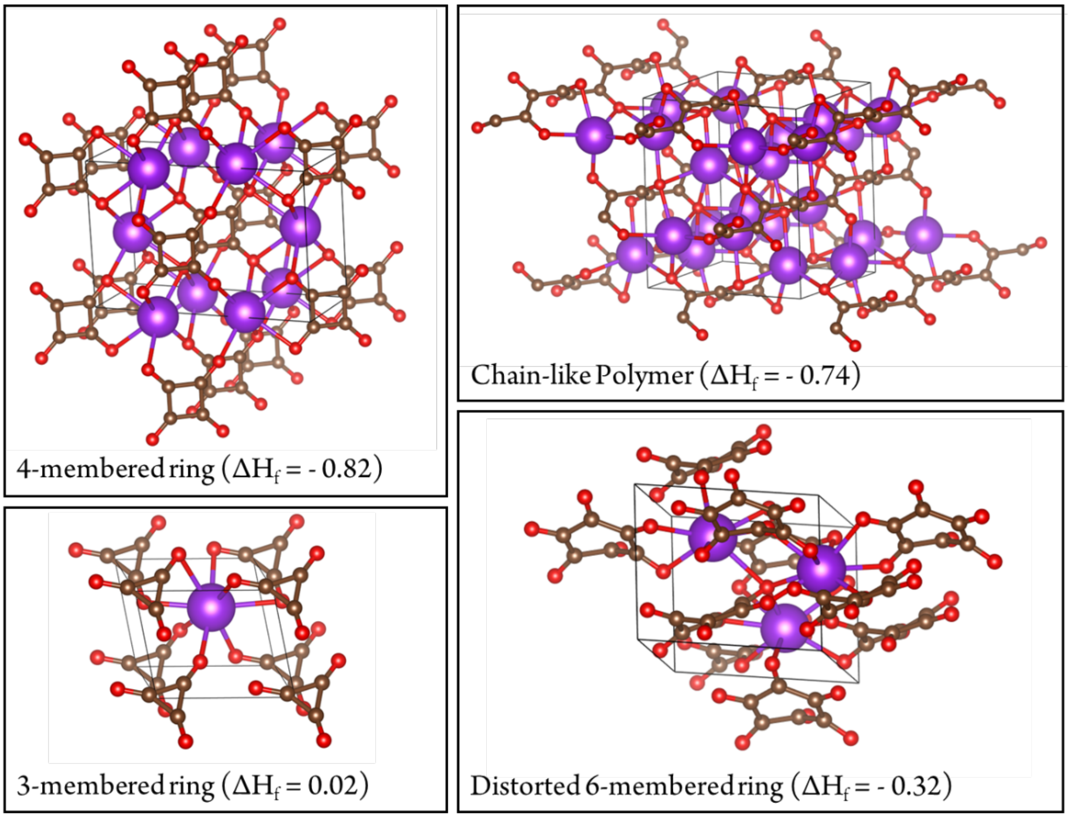

Figure S2. Selected examples of metastable structures found during the K-CO structure search. Formation enthalpies (ΔH_f) are given in eV/f.u.

S.5. Density of States of Selected Low-Energy Phases

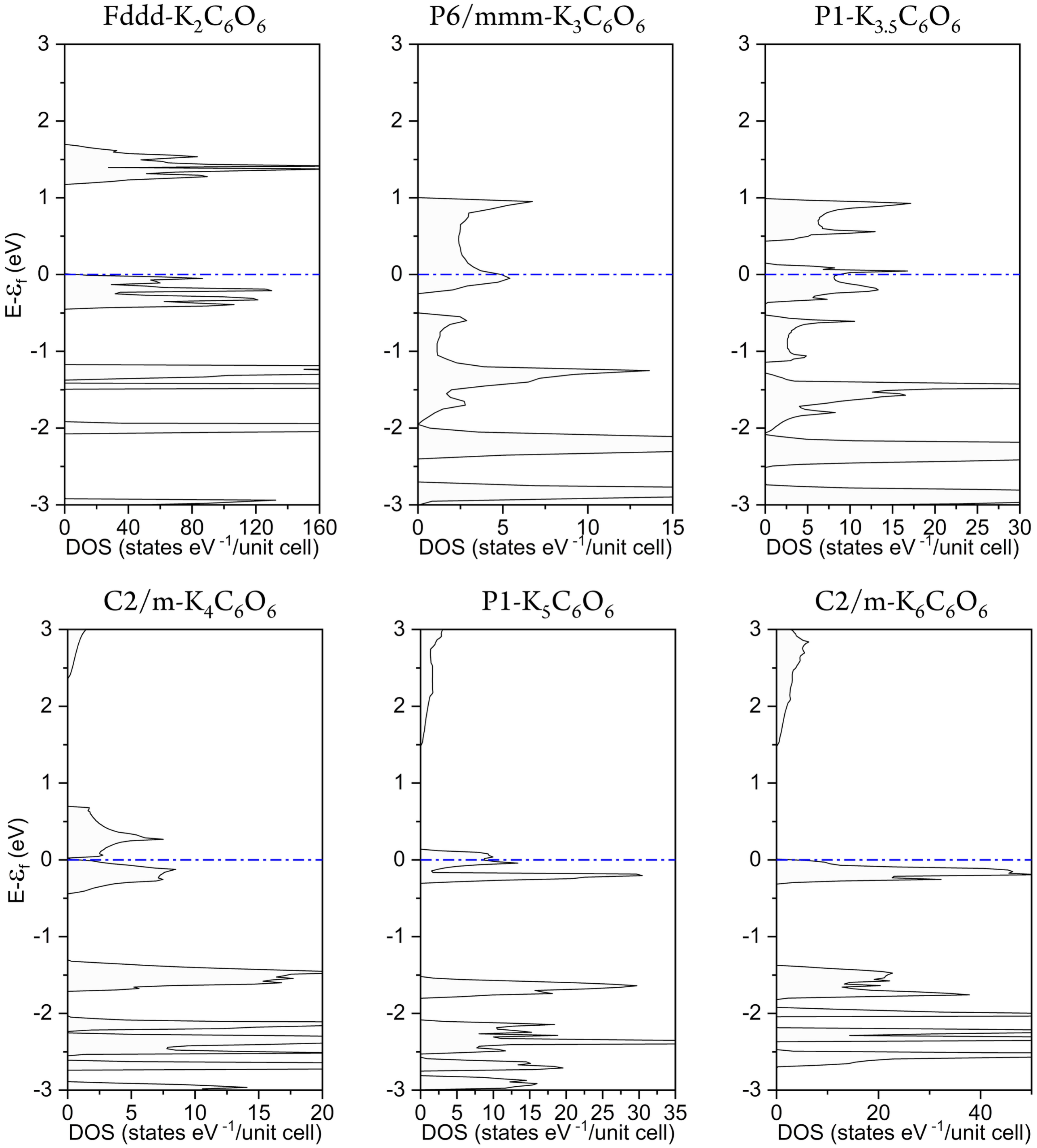

Figure S3. Density of states for the states featured on the K-CO convex hull.

S.6. Phonon Spectra of Selected Low-Energy Phases

P6/mmm phase of $K_3C_6O_6$

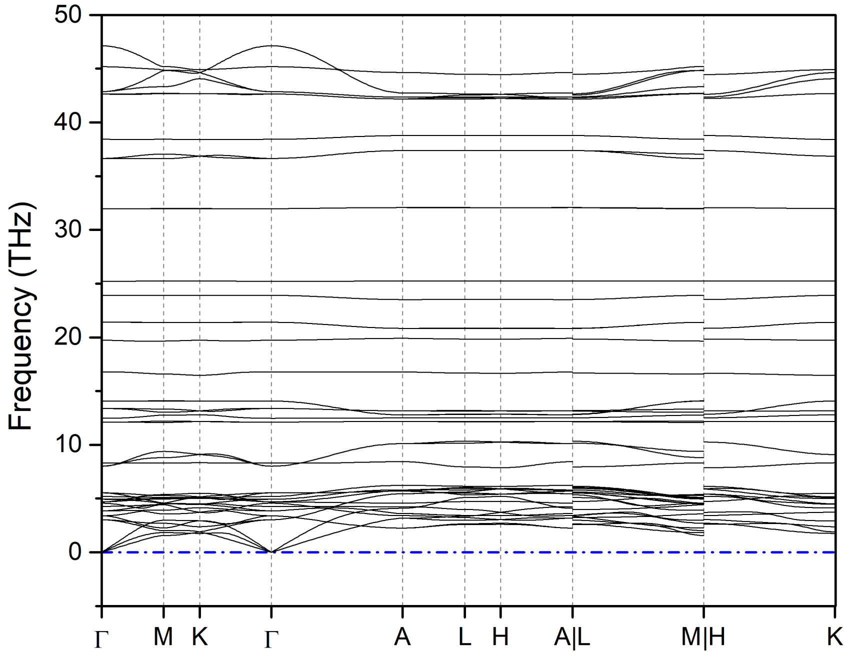

Figure S4. Phonon dispersion curves for the predicted P6/mmm phase of $K_3C_6O_6$, the focus of this work.

P1 phase of $K_{3.5}C_6O_6$.

Due to the low symmetry of this phase, computation of its phonon spectra proved too costly. The 2x2x2 supercell of this phase contains 294 atoms.

C2/m phase of $K_4C_6O_6$

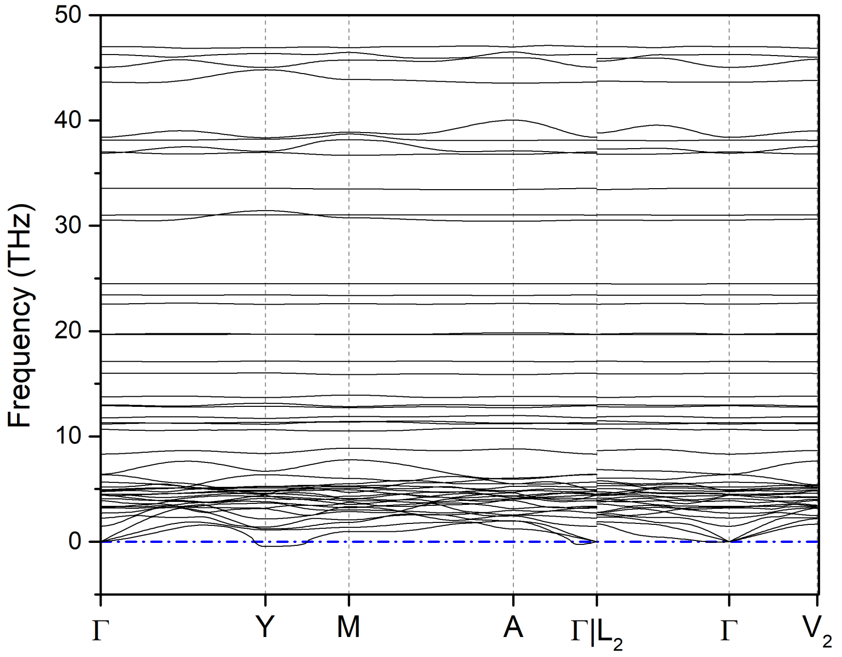

Figure S5a. Phonon dispersion curves for the predicted C2/m phase of $K_4C_6O_6$. Imaginary frequencies are a signal an instability.

Figure S5b shows a distortion of the predicted C2/m phase of $K_4C_6O_6$ obtained by following the Y-point phonon eigenvector with the largest imaginary frequency. The resulting 2×2×1 supercell has *$P2_1/c$* symmetry and is lower in energy by ~1 meV per formula unit relative to the undistorted *C2/m* structure. A full phonon calculation for the *$P2_1/c$* supercell is computationally prohibitive. In any case,

both *C2/m* and the modulated *P2₁/c* variant lie above the convex hull in Figure 1 and are therefore not ground-state phases (unstable or, at best, metastable); as such, they do not affect the conclusions of this work.

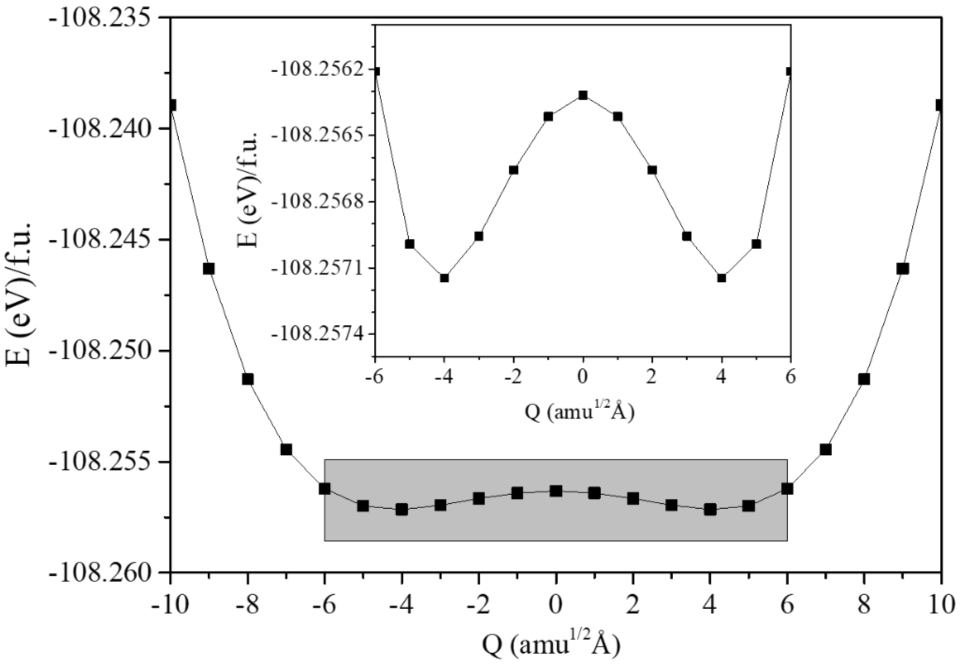

Figure S5b. Energy profile along the greatest frequency imaginary mode of the predicted C2/m phase of $K_4C_6O_6$. The modulation corresponds to a symmetry lowering C2/m → $P2_1/c$.

P1 phase of $K_5C_6O_6$

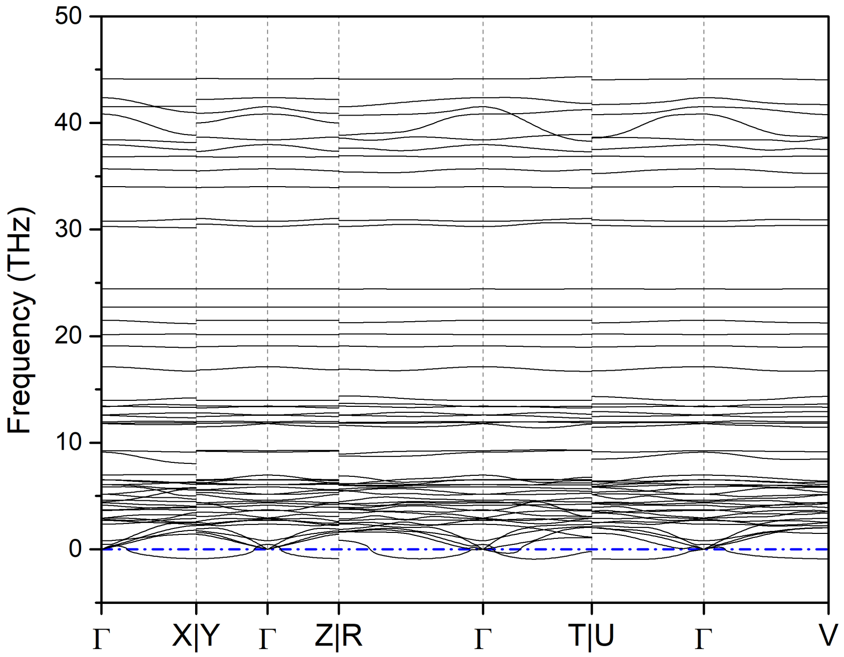

Figure S6. Phonon dispersion curves for the predicted *P1* phase of $K_5C_6O_6$. Imaginary frequencies indicate dynamical instability. Although this phase lies near the convex hull in Figure 1, we did not identify a symmetry-lowered modulation at the same stoichiometry that removes these instabilities. Any such hypothetical stable variant would need to be substantially (-0.32 eV/f.u.) lower in energy than the unstable *P1* structure to affect the predicted stability of $K_3C_6O_6$ and $K_{3.5}C_6O_6$.

C2/m phase of $K_6C_6O_6$

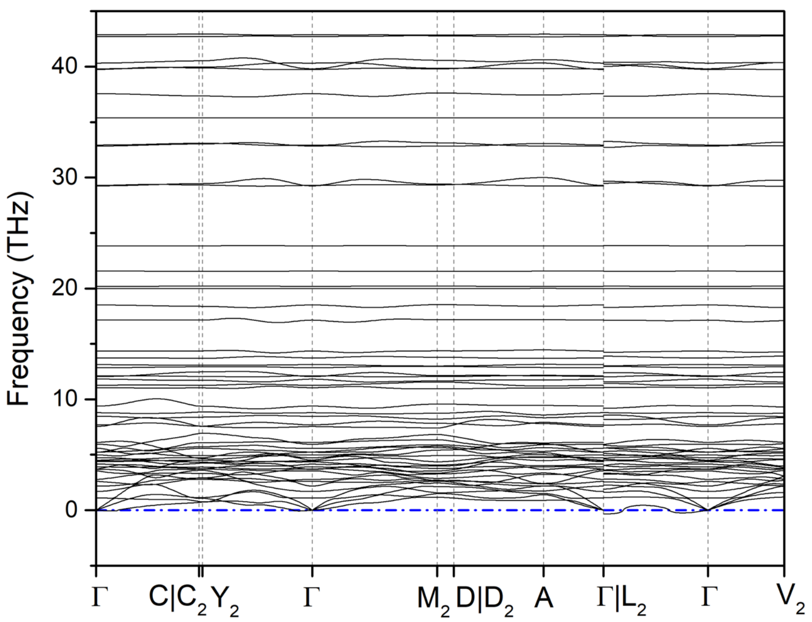

Figure S7. Phonon dispersion curves for the experimental C2/m phase of $K_6C_6O_6$. Imaginary frequencies near Γ are likely due to numerical error.

S.7. Partial charges of atoms and molecular fragments in identified structures

Table S1. Partial charges of atoms and molecular fragments in identified ground state structures.[a]

	$K_2C_6O_6$ (*Fddd*)	$K_3C_6O_6$ (*P6/mmm*)	$K_{3.5}C_6O_6$ (*P1*)	$K_4C_6O_6$ (*C2/m*)	$K_5C_6O_6$ (*P1*)	$K_6C_6O_6$ (*C2/m*)
K	+0.87	+0.86	+0.84	+0.84	+0.83	+0.81
C	+0.83	+0.74	+0.70	+0.58	+0.49	+0.42
O	-1.12	-1.16	-1.19	-1.22	-1.23	-1.23
(C_6O_6)	-1.74	-2.58	-2.94	-3.84	-4.44	-4.86

[a] predicted from the topology of the electron density using the quantum theory of atoms in molecules (QTAIM).

S.8. Density of States for the P6/mmm phase of $K_3C_6O_6$

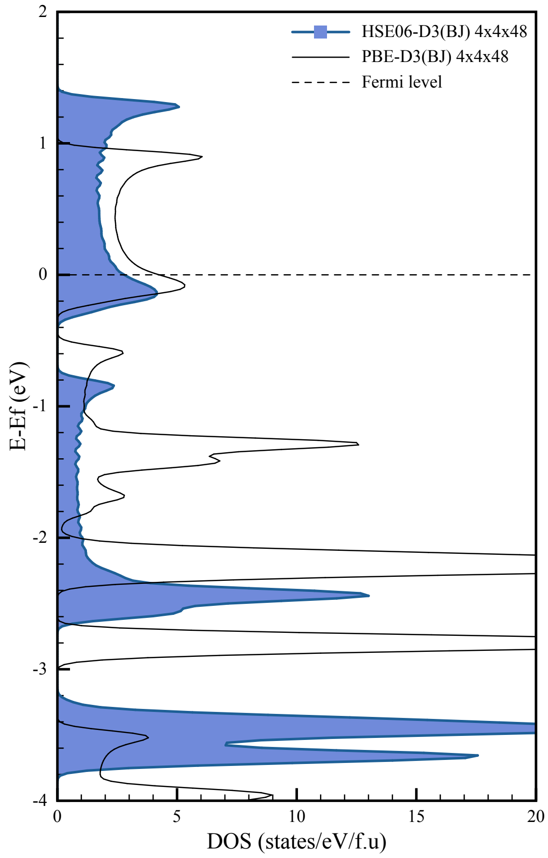

Figure S8. DOS of the P_6/mmm phase of $K_3C_6O_6$, calculated with HSE06 (filled blue) and PBE (black). Each DOS has been calculated for unit cell parameters optimized at the respective level of theory. A noticeably larger dispersion of the ¼-occupied frontier π-band with HSE06 is attributed to a more accurate account of electron correlation, necessary for describing increased electron localization and long-range effects compared to PBE. The number of states at the HSE06 and PBE levels are 2.8 and 4.0 states/eV/formula unit, respectively. The latter value changes only marginally if a HSE06-D3(BJ) geometry is used for both calculations.

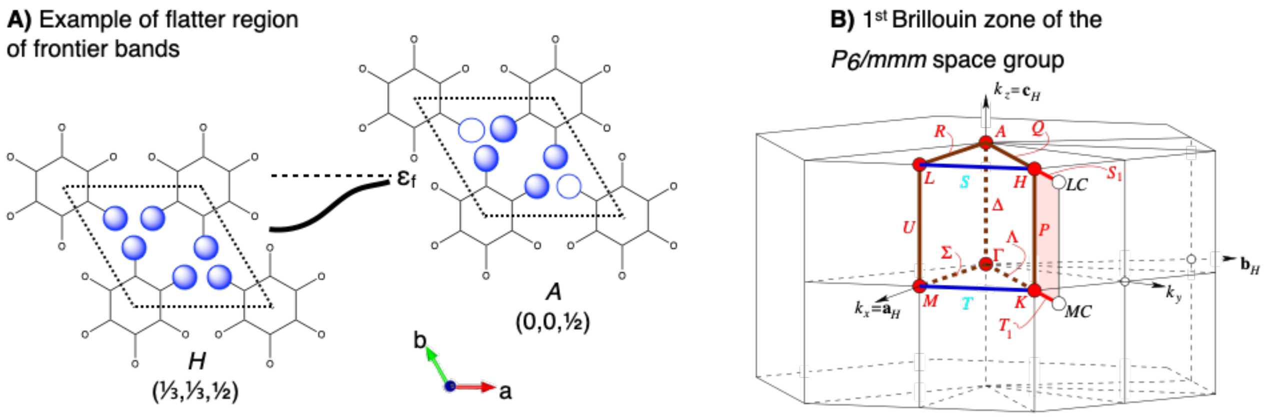

Figure S9. A) Example sketch of weaker orbital interactions between stacks of rhodizonates that explain flatter region of the frontier bands. K^+ ions are omitted for clarity. B) Labels of special points in the 1st Brillouin zone of the P_6/mmm space group.

S.9 Inter-ring distances and van der Waals Radii Comparison in the P6/mmm phase of $K_3C_6O_6$

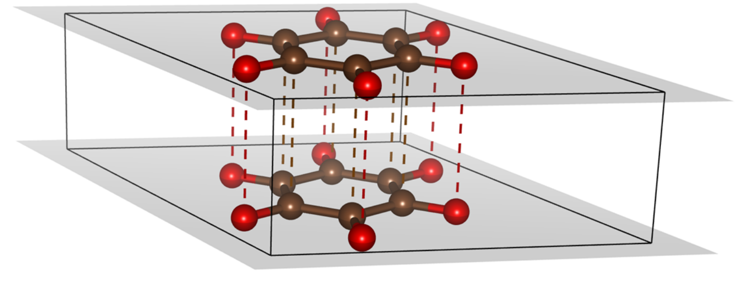

PBE: $d_{OO} = d_{CC} = 3.37$ Å
HSE: $d_{OO} = d_{CC} = 3.34$ Å

Radii	C (Å)	O(Å)
Alvarez[3]	1.77	1.50
Rahm[4]	1.90	1.71
Bondi[5]	1.70	1.52

Figure S10. Interring distances between two $(C_6O_6)^{3-}$ anions in the P6/mmm phase of $K_3C_6O_6$ along with a table with the van der Waals radii from different literature sources.

S.10 1D Inter-ring distance potential Energy Surface

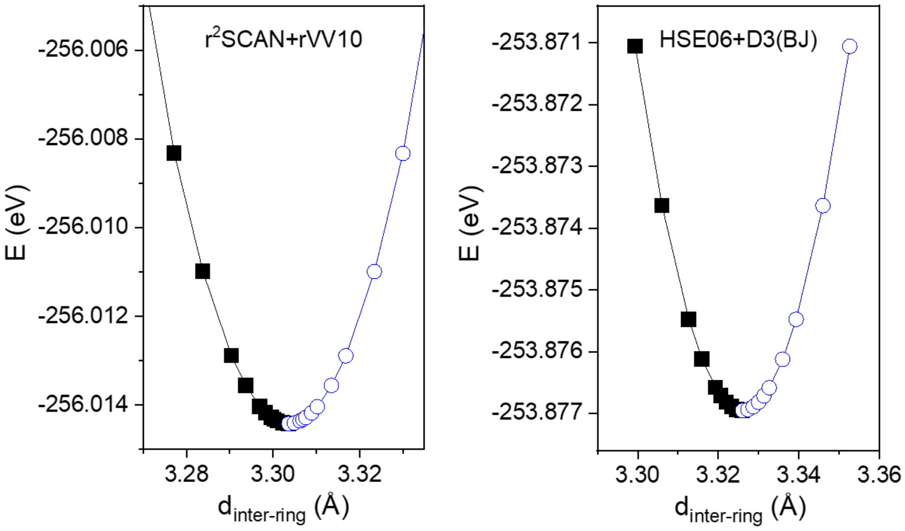

Figure S11.1D Energy profile at different interring distances between two $(C_6O_6)^{3-}$ anions in the *P6/mmm* phase of $K_3C_6O_6$ in a 1x1x2 supercell. Black squares and blue circles correspond to the shortest and longest interring distances in the unit cell.

S.11. Convergence of T_c with respect to thermal smearing

Table S2. Electron phonon coupling (λ), superconducting critical temperature (T_c), average logarithmic frequency (ω_{log}) and BCS superconducting gap at different values of electronic smearing in the energy conserving delta functions of the *P6/mmm* phase of $K_3C_6O_6$

Smearing (eV)	λ	T_c (K)	ω_{log} (meV)	BCS Gap (meV)
0.005	0.327	0.49	39.76	0.074
0.01	0.341	0.68	39.76	0.103
0.02	0.258	0.04	40.39	0.006
0.03	0.245	0.02	40.81	0.003
0.04	0.256	0.04	41.22	0.005

S.12. Computational Details

Structure Prediction: Structure searches were performed using a particle swarm optimization (PSO) algorithm[6] implemented in the Crystal structure Analysis by Particle Swarm Optimization (CALYPSO) software.[7] Our search considered K:CO stoichiometries 2:1, 1:1, 1:2, 1:3 1:4, 2:3, 5:6, 5:12 and 7:12. For most stoichiometries, we performed a scan over the number of formula units per unit cell, ranging from 1 to 6. The exceptions are the larger 5:6, 5:12 and 7:12 ratios, where only one formula unit was considered. For each structure prediction, 30 generations of 30 structures were generated. 60 % of these structures were generated by PSO, while 40% were created by random placement of atoms. In total, we have structurally optimized approximately 35000 unit cells.

Electronic Structure Theory: Structural relaxations were performed using periodic DFT implemented in VASP version 6.1.0.[8-9] For structure prediction we relied on the Predew–Burke–Ernzerhof (PBE) generalized gradient approximation (GGA) exchange correlation functional[10] combined with a D3(BJ) dispersion correction.[11-12] Numerical integrations were made over Gamma-centered *k*-meshes with a reciprocal space resolution < 2π·0.016 Å^{-1}. Our calculations relied on hard projector-augmented wave (PAW) pseudopotentials,[13-14] that include the following valence electrons : K ($3s^23p^64s^1$), C ($2s^22p^2$), O ($2s^22s^4$). The plane wave basis set expansion was limited to 900 eV. Convergence criteria for electronic energies and forces were 10^{-8} eV and 1 meV/Å, respectively. The structure of $K_3C_6O_6$ was additional investigated using the Heyd–Scuseria–Ernzerhof (HSE06) screened hybrid functional, [15-16] combined with the D3(BJ) dispersion correction, in which case we relied on standard PAW potentials for C and O.

Dynamic Stability: Phonon spectra were calculated for the lowest energy phases of $K_nC_6O_6$ (where n=3, 4, 5 and 6) using the finite displacement (2x2x2) supercell method. Finite displacements were calculated with the Parlinski–Li–Kawazoe methodology[17] implemented in PHONOPY version 2.10.[18] These calculations relied on k-mesh densities ≤ 2π·0.03 Å^{-1}, and a kinetic energy cutoff of 800 eV. While no imaginary frequencies were found for $K_3C_6O_6$, small ones, attributable to numerical error, are present for $K_6C_6O_6$. In contrast, the lowest energy phases of $K_4C_6O_6$ and $K_5C_6O_6$ proved dynamically unstable. The discussed $K_{3.5}C_6O_6$ phase is unfortunately of too low symmetry and has a too large unit cell to be feasibly calculated by us (the super cell contains 294 atoms). Phonon spectra are reproduced in Figures S4-S7.

Chemical Bonding Analysis: Crystal Orbital Hamilton Population (COHP) analysis was carried out using LOBSTER version 4.1.0[19-23] with projections of VASP-generated plane wave wavefunctions onto PBEVaspFit2015 atomic orbital basis sets. To estimate the overall bonding contribution between rings, we summed all projected COHP interactions between atoms on one C_6O_6 ring and those on the next ring along the c-axis. The integrated COHP (ICOHP) was then obtained by integrating the resulting curves up to the Fermi level.

We emphasize that a ICOHP value is not a total inter-ring interaction energy in the thermodynamic sense, such as a cohesive energy. Rather, it captures the bonding character of the occupied bands, resolved between selected atom pairs or fragments. It can be interpreted as a semi-quantitative estimate of bonding strength, useful for comparing different stacking geometries or compositions.

As with any energy-decomposition schemes in periodic systems, the ICOHP is not uniquely defined. It depends on the choice of basis set, projection scheme, and the spatial partitioning of orbital interactions. Nonetheless, ICOHP values have proven effective in identifying bonding trends, and here they help quantify the stabilizing effect of π-orbital delocalization along the stacking axis.

Topological analyses of electron densities were made with Critic2.[24] Electron density and volume integrations relied on the Yu-Trinkle algorithm.[24-25]

Superconductivity: Calculations were performed at the PBE-D3(BJ) level with a kinetic energy cutoff of 120 Ry (ca 1632 eV), on a geometry relaxed with Quantum ESPRESSO version 7.3[26] with PAW potentials comparable to those used in VASP. Electronic energies were converged to 10^{-12} Ry and forces to 10^{-5} a.u. For structural relaxation we used a 12x12x24 Monkhorst-Pack *k*-mesh[27] with first order Methfessel-Paxton smearing of 0.005 Ry (~0.068 eV). Phonons were computed using density-functional perturbation theory on a 4x4x8 *q*-mesh.

Electron-phonon quantities were calculated with EPW version 5.5,[29-30] using maximally localized Wanner functions to interpolate electronic bands and electron-phonon matrix elements onto 8x8x16 *k*- and *q*-point grids. The Eliashberg spectral function $\alpha^2F(\omega)$ and coupling constant λ were computed, and T_c was estimated with the McMillan–Allen–Dynes approximation to isotropic Migdal–Eliashberg

theory, using a Coulomb pseudopotential $\mu^* = 0.10$. We assessed convergence of T_c, λ, and the superconducting gap with respect to the electronic smearing σ (Table S2): T_c ranges from ~0.5 K at σ = 0.005 eV to ~0.04 K at σ = 0.04 eV. This variation partly reflects the interplay between smearing, Brillouin-zone sampling, and resolution of Fermi-surface features in electron–phonon calculations.[28] While smaller σ values resolve $\alpha^2F(\omega)$ more sharply, they require correspondingly denser k/q grids. At σ = 0.005 eV the grid densities we could afford are likely insufficient for full convergence, so the reported T_c values carry a corresponding uncertainty.

S.11. References

[1] J. Sun, D. D. Klug, C. J. Pickard, R. J. Needs, *Phys. Rev. Lett.* **2011**, *106*, 145502.
[2] K. Xia, J. Sun, C. J. Pickard, D. D. Klug, R. J. Needs, *Phys. Rev. B* **2017**, *95*, 144102.
[3] S. Alvarez, *Dalton Trans* **2013**, *42*, 8617-8636.
[4] M. Rahm, M. Ångqvist, J. M. Rahm, P. Erhart, R. Cammi, *ChemPhysChem* **2020**, *21*, 2441-2453.
[5] A. Bondi, *J. Phys. Chem.* **1964**, *68*, 441-451.
[6] Y. Wang, J. Lv, L. Zhu, Y. Ma, *Phys. Rev. B* **2010**, *82*, 094116.
[7] Y. Wang, J. Lv, L. Zhu, Y. Ma, *Comput. Phys. Commun.* **2012**, *183*, 2063-2070.
[8] G. Kresse, J. Furthmüller, *Phys. Rev. B* **1996**, *54*, 11169-11186.
[9] G. Kresse, J. Furthmüller, *Comput. Mater. Sci.* **1996**, *6*, 15-50.
[10] J. P. Perdew, K. Burke, M. Ernzerhof, *Phys. Rev. Lett.* **1996**, *77*, 3865-3868.
[11] S. Grimme, J. Antony, S. Ehrlich, H. Krieg, *J. Chem. Phys.* **2010**, *132*, 154104.
[12] S. Grimme, S. Ehrlich, L. Goerigk, *J. Comput. Chem.* **2011**, *32*, 1456-1465.
[13] P. E. Blöchl, *Phys. Rev. B* **1994**, *50*, 17953-17979.
[14] G. Kresse, D. Joubert, *Phys. Rev. B* **1999**, *59*, 1758-1775.
[15] A. V. Krukau, O. A. Vydrov, A. F. Izmaylov, G. E. Scuseria, *J. Chem. Phys.* **2006**, *125*, 224106.
[16] J. Heyd, G. E. Scuseria, M. Ernzerhof, *J. Chem. Phys.* **2003**, *118*, 8207-8215.
[17] K. Parlinski, Z. Q. Li, Y. Kawazoe, *Phys. Rev. Lett.* **1997**, *78*, 4063-4066.
[18] A. Togo, I. Tanaka, *Scr. Mater.* **2015**, *108*, 1-5.
[19] R. Dronskowski, P. E. Bloechl, *J. Phys. Chem.* **1993**, *97*, 8617-8624.
[20] V. L. Deringer, A. L. Tchougréeff, R. Dronskowski, *J. Phys. Chem. A* **2011**, *115*, 5461-5466.
[21] S. Maintz, V. L. Deringer, A. L. Tchougréeff, R. Dronskowski, *J. Comput. Chem.* **2013**, *34*, 2557-2567.
[22] R. Nelson, C. Ertural, J. George, V. L. Deringer, G. Hautier, R. Dronskowski, *J. Comput. Chem.* **2020**, *41*, 1931-1940.
[23] S. Maintz, V. L. Deringer, A. L. Tchougréeff, R. Dronskowski, *J. Comput. Chem.* **2016**, *37*, 1030-1035.
[24] A. Otero-de-la-Roza, E. R. Johnson, V. Luaña, *Comput. Phys. Commun.* **2014**, *185*, 1007-1018.
[25] M. Yu, D. R. Trinkle, *J. Chem. Phys.* **2011**, *134*, 064111.
[26] P. Giannozzi, S. Baroni, N. Bonini, M. Calandra, R. Car, C. Cavazzoni, D. Ceresoli, G. L. Chiarotti, M. Cococcioni, I. Dabo, A. Dal Corso, S. de Gironcoli, S. Fabris, G. Fratesi, R. Gebauer, U. Gerstmann, C. Gougoussis, A. Kokalj, M. Lazzeri, L. Martin-Samos, N. Marzari, F. Mauri, R. Mazzarello, S. Paolini, A. Pasquarello, L. Paulatto, C. Sbraccia, S. Scandolo, G. Sclauzero, A. P. Seitsonen, A. Smogunov, P. Umari, R. M. Wentzcovitch, *J. Phys. Condens. Matter* **2009**, *21*, 395502.
[27] H. J. Monkhorst, J. D. Pack, *Phys. Rev. B* **1976**, *13*, 5188-5192.
[28] M. Wierzbowska, S. de Gironcoli, P. Giannozzi, *arXiv preprint cond-mat/0504077* **2005**.
[29] F. Giustino, M. L. Cohen, S. G. Louie, *Phys. Rev. B* **2007**, *76*, 165108.
[30] S. Poncé, E. R. Margine, C. Verdi, F. Giustino, *Comput. Phys. Commun.* **2016**, *209*, 116-133.
